# The Preferential Impairment of Pupil Constriction Stimulated by Blue Light in Patients with Type 2 Diabetes without Autonomic Neuropathy

**DOI:** 10.1155/2017/6069730

**Published:** 2017-03-22

**Authors:** Fukashi Ishibashi, Rie Kojima, Miki Taniguchi, Aiko Kosaka, Harumi Uetake, Mitra Tavakoli

**Affiliations:** ^1^Ishibashi Clinic, Hiroshima, Japan; ^2^University of Exeter Medical School, Exeter, UK

## Abstract

The main aim of the present paper is to examine whether the pupillary light reflex (PLR) mediated by intrinsically photosensitive retinal ganglion cells (ipRGCs) is impaired in type 2 diabetic patients. One hundred and three diabetic patients without diabetic autonomic neuropathy (DAN) and 42 age-matched controls underwent a series of detailed neurological examinations. The patients were stratified into three groups: stage I, no neuropathy; stage II, asymptomatic neuropathy; stage III, symptomatic but without DAN. The PLR to 470 and 635 nm light at 20 cd/m^2^ was recorded. Small fiber neuropathy was assessed by corneal confocal microscopy and quantifying corneal nerve fiber (CNF) morphology. The 470 nm light induced a stronger and faster PLR than did 635 nm light in all subjects. The PLR to both lights was impaired equally across all of the diabetic subgroups. The postillumination pupil response (PIPR) after 470 nm light offset at ≥1.7 sec was attenuated in diabetic patients without differences between subgroups. Receiver operating characteristic analysis revealed that the PIPR mediated by ipRGCs in patients with stage II and stage III neuropathy was different from that of the control subjects. Clinical factors, nerve conduction velocity, and CNF measures were significantly correlated with PLR parameters with 470 nm light. PLR kinetics were more impaired by stimulation with blue light than with red light in diabetic patients without DAN.

## 1. Introduction

Pupillary dysfunction is considered to be an early sign of systemic autonomic neuropathy [1]. The pupillary light reflex (PLR) is maintained in humans who are blind because of extrinsic outer retinal damage, indicating the presence of intrinsically photosensitive cells in the retina [2]. The rods-cones and melanopsin-expressing intrinsically photosensitive retinal ganglion cells (ipRGCs) operate together to regulate the PLR [3]. The ipRGCs are the third photoreceptor cells in the human and primate eye [4]. The primary function of ipRGCs is non-image-forming photoreception, for mediating the PLR via signaling to the pretectal olivary nucleus [5] and for the signaling of environmental irradiation to entrain the central body clock to the solar day to maintain the circadian rhythm. Red light produces pupil constriction mediated by cone input via transsynaptic activation of melanopsin-expressing retinal ganglion cells (RGCs), whereas blue light leads to pupil constriction mediated primarily by direct photoactivation of ipRGCs. Rods and cones have a physiological role in the PLR, and ipRGCs receive synaptic signals from the outer retina. The relative contributions of cones-rods and ipRGCs change depending on the stimulus wavelength, irradiance strength, and temporal profile [6, 7]. The contribution of melanopsin can only be isolated after the offset of a long duration of light irradiation as the postillumination pupil response (PIPR) [4]. Therefore, it is not possible to exactly determine the relative contributions of rods-cones and ipRGCs even when blue and red stimuli are employed for examining PLR kinetics. However, the PLR to chromatic stimuli is the only measurable, noninvasive physiological response that directly reflects the cumulative behavior of the three types of retinal photoreceptor [8, 9]. Recently, Adhikari et al. reported that, after a 1 sec light pulse, PIPRs at <1.7 sec and ≥1.7 sec are best described by the actions of the combination of ipRGCs and rods and solely of ipRGCs, respectively [10].

The PLR has been used to diagnose diabetic autonomic neuropathy (DAN) [11, 12]. Apart from one case series [13], there have been no large-scale investigations in diabetic patients examining the relative contributions of the inner and outer photoreceptors selectively using the PLR induced by chromatic light. Of course, the PLR is the results of a neural reflex that is dependent upon pathways and synaptic events beyond the retina. The correlations between the parameters of PLR kinetics and clinical factors, neurophysiological tests, and CNF measures might indicate the disturbance of PLR arc beyond the retinal photoreceptors. The contribution of clinical factors other than hyperglycemia in impaired PLR in diabetic patients stimulated by blue and red light has never been investigated.

The present study aimed to measure the PLR stimulated by chromatic light as a differential assessment of the inner and outer retinal function and to clarify the possible causative role of the dysfunction of ipRGCs in the impaired PLR using commercially available equipment in a large number of type 2 diabetic patients without DAN.

## 2. Research Design and Methods

### 2.1. Subjects

Between June 2014 and November 2015, 103 Japanese patients with type 2 diabetes without clinical evidence of DAN as assessed by detailed examinations of diabetic neuropathy as defined in the Diabetic Neuropathy Study Group in Japan (DNSGJ) have been enrolled [14], at the Ishibashi Clinic, Hiroshima, Japan. 42 age-matched healthy subjects (HbA1c <5.7%, and fasting plasma glucose <5.5 mM or casual postprandial plasma glucose <7.7 mM) were recruited as control group. The exclusion criteria were as follows: being older than 55 years (because of the potential for yellowing of the crystalline lens [15]), color blindness, proliferative or preproliferative diabetic retinopathy, other retinal or ocular diseases, wearing hard (Rigid Gas Permeable) contact lenses, neurodegenerative diseases, and taking any drugs that affect autonomic nerve functions. Written informed consent was obtained from all subjects. The ethics committee of the Ishibashi Clinic approved the protocol of the study. All participants underwent detailed clinical, neurological, and ophthalmic assessments.

Gender and age were similar between the control group and the diabetic group and between the diabetic subgroups stratified by neuropathy severity (Table 1).

### 2.2. Ophthalmic Examinations

#### 2.2.1. Pupillary Light Reflex (PLR)

**Figure 1:**
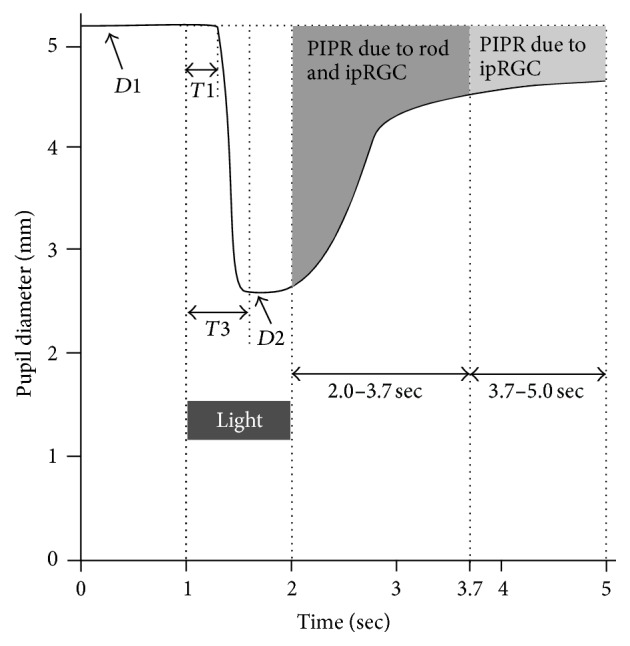
Pupillary light reflex waveform and kinetic parameters.* D*1, baseline average pupil diameters for one sec before light stimulus (470 nm or 635 nm);* T*1, period required to start pupil constriction after light stimulus;* D*2, the minimum pupil diameter;* T*3, period for* D*2 after light stimulus; PIPR (postillumination pupillary response) after light offset, due to rod and ipRGC at <1.7 sec and due to ipRGC at ≤1.7–3.0 sec after light offset.

After dark adaptation for 10 min in a dark room, blue (470 nm) or red (635 nm) light of 20 cd/m^2^ was emitted for 1 sec to the right or left eye in a random order, and changes in pupil diameter of bilateral eyes were recorded simultaneously using Iris corder Dual C10641 equipment (Hamamatsu Photonics Inc., Hamamatsu, Shizuoka, Japan) for 5 seconds. 470 nm light as a blue light and 635 nm light as a red light have the highest spectral powers. At 20 cd/m^2^, 470 nm and 635 nm light are equivalent to 4.79 × 14 log and 2.14 × 14 log photons/cm^2^/sec, respectively, when the pupil diameter is assumed to be 6.0 mm. We used red and blue stimuli in a random order but could not examine the influence of previous light exposure on the amplitude of pupil constriction caused by the next light exposure, because previous exposure to long wavelength light increases the amplitude of pupil constriction, whereas short wavelength light decreases it [16]. However, we used weak light irradiance for short periods (1 sec), so the influence of previous light exposure should have been small. The period between two stimuli was 15 min, to allow the pupil diameter to return to the baseline level. The light pulses were projected within the housing of a pair of goggles. The dark-adapted (baseline) pupil diameter (mm,* D*1), minimum pupil diameter (mm,* D*2) after light emission, pupil diameter constriction (*D*1 −* D*2, mm, PC), latency period (time required to start pupil constriction after light stimulus, msec,* T*1), period required for* D*2 (msec,* T*3), and velocity of constriction ([*D*1 −* D*2]/*T*3, *μ*/msec, CS) were calculated automatically by the apparatus. The PIPR was arbitrarily estimated from the area under the curve (AUC) by counting pixel numbers. According to a recent report by Adhikari et al. [10], AUCs of <1.7 sec and ≥1.7 to 3.0 sec are assumed to result from rod and melanopsin signaling and solely melanopsin signaling, respectively (Figure 1). Four measurements conducted for bilateral light-stimulated and consensual eyes were averaged. For eliminating the relative unilateral afferent pupil defects in patients, we excluded the patients whose PLR kinetic parameters were apparently different between bilateral eyes. All subjects were tested between 9 and 12 am [17].

#### 2.2.2. Corneal Confocal Microscopy (CCM)

All subjects were examined using a Heidelberg Retina Tomograph 3 equipped with a Rostock Cornea Module (Heidelberg Engineering, Heidelberg, Germany) [18]. Six high-clarity images per subject were analyzed to quantify the following parameters, to quantify the corneal nerve fibers (CNFs): CNF density (CNFD), the total number of major nerve fibers/mm^2^; CNF length (CNFL), the total length of major nerve fibers (mm/mm^2^); corneal nerve branch density (CNBD), the number of branches emanating from all major nerve trunks/mm^2^; corneal nerve branch length (CNBL), the total length of the corneal nerve branch (mm/mm^2^); tortuosity grade (TG); frequency/0.1 mm of beading (BF); and bead size (BS, *μ*m^2^). Except for the TG and BS, all measurements were performed using ImageJ (Texelcraft, Tokyo, Japan); the TG was measured using the criteria of Oliveira-Soto and Efron [19], and the BS was determined as previously reported [20].

### 2.3. Assessment of Neuropathy

Diabetic neuropathy was assessed in type 2 diabetic patients according to the simplified diagnostic criteria proposed by the DNSGJ [14], based on the presence of two of the following three factors: subjective symptoms in the bilateral lower limbs or feet, absent or reduced ankle jerk, and decreased vibration perception. The diabetic patients were classified into one of five stages of diabetic neuropathy as defined in the DNSGJ criteria [14]: stage I, without diabetic neuropathy; stage II, asymptomatic diabetic neuropathy; stage III, symptomatic but either the ankle jerk reflex or vibration sensation was normal; stage IV, with autonomic neuropathy; and stage V, with motor neuropathy. Patients with stage IV or V neuropathy were excluded from the present study.

### 2.4. Neurophysiological Examinations

Electrophysiology and nerve conduction velocity (NCV) studies were performed using an electromyography instrument (Neuropack S1, Nihon Kohden, Tokyo, Japan) for the median nerve (motor) and the ulnar and sural nerves (sensory).

The vibration perception threshold (VPT) was measured at the left medial malleolus using a biothesiometer (Biomedical Instruments, Newbury, OH, USA). The warm and cold perception thresholds (PTs) at the dorsum of the foot were determined using a thermal stimulator (Intercross-200, Intercross Co., Tokyo, Japan). To assess cardiovagal function, the coefficient of variation in R-R intervals (CV_R-R_) was calculated from the R-R intervals of 200 samples on an electrocardiogram.

### 2.5. Medical and Laboratory Data

Body mass indexes (BMIs) and blood pressures were determined (Table 1). Glycated hemoglobin (HbA1c) levels were converted to National Glycohemoglobin Standardization Program (NGSP) units by adding 0.4% to the measured values [21]; they were subsequently converted to International Federation of Clinical Chemistry values by using the equation [(10.93NGSP)–23.50]. Serum creatinine levels, lipid profiles, urinary albumin creatinine ratio (ACR), and estimated glomerular filtration rate (eGFR) were also determined.

### 2.6. Statistical Analyses

All statistical analyses were performed using SPSS version 19 (SPSS, Chicago, IL, USA). All values are presented with 95% confidence intervals (CIs). All data sets were tested for normality using the Shapiro-Wilk test. Comparisons between subjects with and without diabetes were made by Student's* t*-test and Mann–Whitney *U* test for normally and nonnormally distributed continuous variables, respectively, and Fisher's exact test for categorical variables. PLR parameters obtained from using 470 nm and 635 nm light were compared in the controls and the patients with type 2 diabetes using Wilcoxon's signed-rank test. Comparisons of normally distributed variables between the control group and the diabetic subgroups were made using one-way analysis of variance (ANOVA) for continuous variables and Fisher's exact test for categorical variables, followed by Bonferroni corrections. For nonnormally distributed variables, the Kruskal-Wallis test was applied with subsequent Mann–Whitney's *U* test and Bonferroni corrections. The diagnostic value of a PIPR at ≥1.7 sec after blue light offset, for differentiating between the control group and the diabetic subgroups, was assessed using receiver operating characteristic (ROC) curve analysis. Multivariate regression analysis was used to determine the relationship between PLR parameters and clinical factors, neurophysiological tests, and CNF measures in the diabetic patients. A *p* < 0.05 was considered significant.

## 3. Results

### 3.1. Clinical Characteristics of Control Subjects and Subgroups of Type 2 Diabetic Patients

The demographic data of the control subjects and diabetic patients are presented in Table 1. The BMIs of the diabetic patients were higher than those of the control subjects. The systolic blood pressure in all of the diabetic subgroups and the diastolic blood pressure in the subgroup with stage III neuropathy were higher than those of the control subjects. Angiotensin receptor blockers were prescribed more frequently for all diabetic patients than for the control subjects. The HbA1c levels in all of the diabetic subgroups were higher than those of the control group, and the HbA1c levels in patients with stage III neuropathy were higher than those in the patients without neuropathy. High-density lipoprotein- (HDL-) cholesterol levels in all of the diabetic subgroups were lower than those in the control group. The triglycerides levels in the subgroup of patients with stage III neuropathy were higher than those of the control subjects. The ACR in the subgroup with stage III neuropathy was higher than that of the control group. The incidence of simple diabetic retinopathy was similar among the diabetic subgroups.

### 3.2. Neurophysiological Tests

Neurophysiological test results in the patients without neuropathy were not different from those of the control subjects (Table 2). The NCV and amplitude of the median and ulnar nerves in the subgroup of patients with stage II neuropathy were lower compared with those of the control subjects and were even lower in patients with stage III neuropathy. The SCV of the sural nerve in patients with stage III neuropathy was slower than that in the control subjects. CV_R-R_ and the temperature PTs in all diabetic subgroups were not significantly different from those in the control group (Table 2).

### 3.3. Corneal Nerve Fiber (CNF) Morphological Parameters in Control Subjects and Diabetic Subgroups

**Figure 2:**
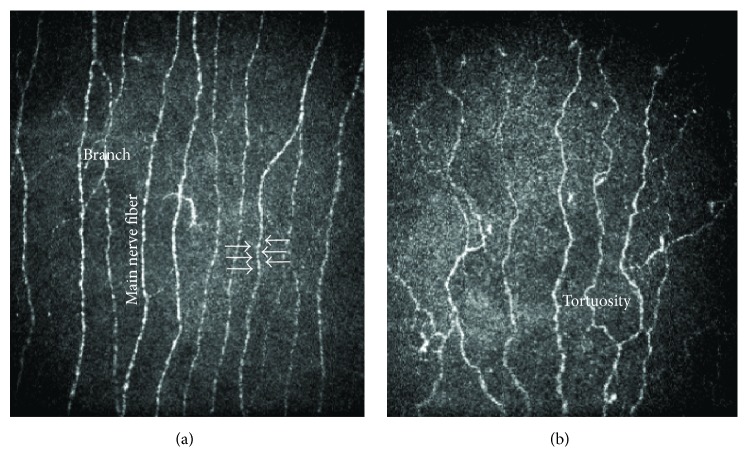
Comparison of the morphology of corneal nerve fibers between a control subject (a) and a patient without neuropathy (b). White arrows indicated beads.

The CNFD and BF in patients without neuropathy were significantly lower, and the TG and BS were higher, compared with those of the control subjects (Table 3). In patients with stage II neuropathy, all CNFs measures except for CNBL were significantly different from those of the control subjects, and CNFL was smaller in patients with stage III neuropathy than in those with stage I neuropathy (Table 3).  Figure 2 compares the CNF morphology between a control subject (a) and a diabetic patient without neuropathy (b). CNFD and CNBD were lower and tortuosity was higher in a patient without neuropathy.

### 3.4. Baseline Pupil Size and PLR Kinetic Parameters in Control Subjects, Total Type 2 Diabetic Patients, and Their Subgroups

**Figure 3:**
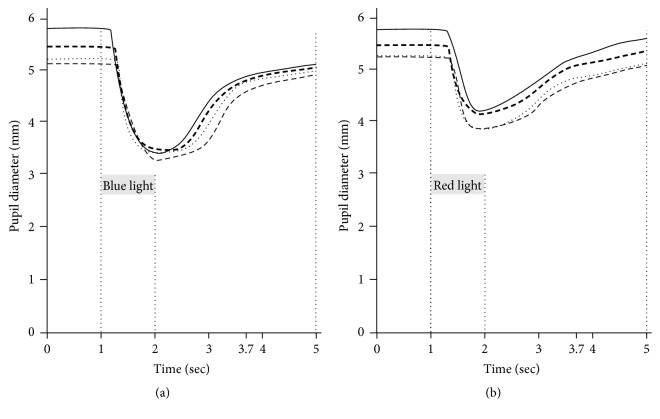
Comparison of average pupillary light reflex waveform on blue light (a) and red light (b) exposure between controls (—) and diabetic subgroups {I; without neuropathy (------), II; asymptomatic neuropathy (········), and III; symptomatic but without diabetic autonomic neuropathy (- - -)} stratified by the severity of neuropathy.

The average PLR waveforms obtained from blue (Figure 3(a)) and red (Figure 3(b)) light exposure were compared among the control group and the diabetic subgroups.* D*1 of the diabetic patient group was smaller than that of the control group (Table 4).* T*1 after 470 nm light exposure was shorter than that after 635 nm light exposure in all subjects, and* T*1 after exposure to both lights was longer in diabetic patients than in control subjects. The PC caused by 470 nm light was larger than that caused by 635 nm light in all subjects, and the PC caused by both stimuli was smaller in diabetic patients than in control subjects. The CS caused by 470 nm light was faster than that caused by 635 nm light in all subjects, and the CS caused by 470 nm light was slower in diabetic patients than in the control subjects. The PIPRs at <1.7 sec and ≤1.7–3.0 sec after blue light offset were larger than those after red light offset in all subjects. The PIPRs at <1.7 sec and ≤1.7–3.0 sec after blue light offset were smaller in the diabetic patients than in the control subjects.

The PLR parameters obtained from exposure to 470 nm and 635 nm light were compared between the control subjects and the diabetic subgroups (Table 4).* D*1 before 470 nm and 635 nm light exposure was smaller in all diabetic subgroups than in the control group but was similar among the subgroups.* T*1 after exposure to 470 nm light was longer in all of the diabetic subgroups than in the control group, but there were no differences among the subgroups.* T*1 after exposure to 635 nm light of patients with stage I neuropathy was longer than that of the control subjects. In all subgroups,* T*1 after exposure to 635 nm light was longer than that after exposure to 470 nm light. The pupil constriction amplitudes in all diabetic patients and subgroups were smaller than those in the control group.* T*3s tended to be shorter in the diabetic patients than in the control subjects, but the differences did not reach significance (*p* = 0.089–0.341). PCs caused by 470 nm light were equally smaller in all subgroups than in the control group, whereas PCs caused by 635 nm light were similar across the control group and diabetic subgroups. Among the diabetic subgroups, the PCs caused by 470 nm light were more intense than those caused by 635 nm light. The CS caused by 470 nm light was slower in all of the diabetic subgroups than in the control group, whereas the CSs caused by 635 nm light were similar among the control group and the diabetic subgroups. The CSs caused by 470 nm light were faster in all of the diabetic subgroups than those caused by 635 nm light. The PIPRs at <1.7 sec and ≤1.7–3.0 sec after blue light offset were larger in the control group than in all of the diabetic subgroups.

### 3.5. ROC Curve Analysis of the PIPR Mediated by ipRGCs after Blue or Red Light Offset

**Figure 4:**
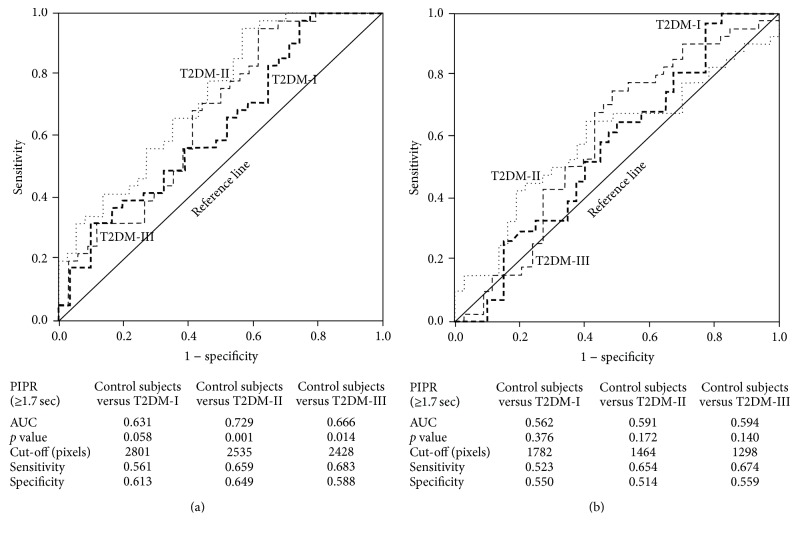
The receiver operating characteristic (ROC) curve analysis of postillumination pupillary response (PIPR) after the offset of blue (a) and red (b) light at ≥1.7–3.0 sec between the control subjects and diabetic subgroups stratified by the severity of neuropathy {I; without neuropathy (------), II; asymptomatic neuropathy (········), and III; symptomatic but without diabetic autonomic neuropathy (- - -)}.

According to ROC curves of the PIPR at ≥1.7 sec after blue (Figure 4(a)) or red light (Figure 4(b)) offset in the control group and the diabetic subgroups, the PIPRs after blue light offset of patients with stage II and stage III neuropathy had the diagnostic value for the dysfunction of ipRGCs in diabetic subgroup with stage II and III, while PIPR after red light offset did not.

### 3.6. Correlations between PLR Parameters and Clinical Factors, Neurophysiological Tests, and CCM Measures

Age was negatively correlated with the* T*1 and CS upon stimulation with 470 nm light and with the PC upon stimulation with lights of both wavelengths (Table 5). Blood pressure was negatively correlated with* D*1. HbA1c level was positively and HDL-cholesterol level was negatively correlated with* T*1 upon stimulation with 470 nm light. The amplitudes of the median nerve, CNFD, CNFL, CNBD, and CNBL were all positively correlated with* D*1 (Table 5). The PIPRs at <1.7 sec and ≥1.7 sec after blue light offset were not significantly correlated with any clinical factors, neurophysiological tests, or CNF measures (*p* = 0.065–0.983).

## 4. Discussion

It is becoming clear that neuroretinal cells [22, 23], especially RGCs [24, 25], are affected in the early stage of diabetes. Since the discovery of ipRGCs, their pivotal role in the PLR and circadian rhythm has been recognized [26, 27]. The ipRGCs project to the olivary pretectal nuclei, constituting the afferent arm of the PLR [28]. Although there were many methodological limitations, we were able to differentially evaluate the functions of cones-rods and ipRGCs using the PLR caused by red and blue light with the sensitive wave length and elucidate which photoreceptors were impaired by diabetes.

When the PLR kinetics following long-standing blue and red light irradiation were compared, the onset of pupil constriction caused by blue light was slower than that caused by red light, and the PIPR after the offset of blue light persisted longer than that after red light, to which the ipRGCs solely contribute [4, 6]. However, the relative contributions of cones-rods and ipRGCs change depending on the stimulus wavelength, irradiation strength, and temporal profile [6, 7]. Therefore, many human PLR studies of ipRGCs have employed stimuli with long durations (>10 sec) [4, 29, 30], which robustly established the contribution of ipRGCs to PLR kinetics. Using 10 sec stimulus with a 30 sec follow-up period after light offset, Feigl et al. demonstrated impaired ipRGC function in diabetic patients for the first time [13]. Park et al. [31] reported that blue light stimulation for 1 sec elicited a prolonged PIPR after light offset more effectively than 10 sec stimulation. Recently, Adhikari et al. [10] elegantly showed that, after a 1 sec light pulse, PIPR spectral sensitivity at ≥1.7 sec after light offset is best described by ipRGC contribution, and at times <1.7 sec the effect is mediated by rods and ipRGCs. These two reports enabled us to use 1 sec chromatic light stimulation to clinically elicit the impaired contribution of ipRGCs to the PLR using a large sample size. We used 470 nm blue light because it has the highest sensitivity in the short wavelength region of the spectrum of light that ipRGCs respond to. Therefore, the present study employed light at 470 nm and 635 nm for 1 sec with a follow-up period after light offset of 3 sec, and the PIPR after light offset was assessed by the area within the redilatation curve at ≥1.7 sec and <1.7 sec separately [10]. This enabled the elucidation of the kinetic differences in PLR caused by two different light stimuli between control subjects, type 2 diabetic patients, and the subgroups of diabetic patients stratified by the severity of neuropathy. Since the melanopsin PIPR can persist for as long as 80 sec [32], our follow-up period of 3 sec was too short to fully elucidate the contribution of ipRGCs. In addition, because we did not perform spectral analysis, the relative contributions by cones-rods and ipRGCs to the early metrics of the PLR could not be assessed. As previously reported [11],* D*1 of the diabetic patients was smaller than that of the control subjects. We did not dilate the pupils and used a low level of light irradiance close to the threshold for eliciting a melanopsin signal. These methodological limitations may have influenced the results of the present study.

In this study, a more rapid and intense pupil constriction was caused by blue light than by red light in control subjects as well as in diabetic patients. Irrespective of the severity of neuropathy, pupil constriction caused by blue and red light in diabetic patients was slower and less pronounced than that in the control subjects.* T*1 after stimulation by both kinds of light was longer in the diabetic patients than in the control subjects. The PIPRs at ≥1.7 sec and <1.7 sec after blue light offset in the control subjects were larger than those after red light offset. The PIPRs at ≥1.7 sec and <1.7 sec after blue light offset were smaller in the diabetic group than in the control subjects, irrespective of the severity of neuropathy. These results indicate that 1 sec irradiation with blue or red light was clinically useful for eliciting different PLR kinetics in the control subjects and patients with type 2 diabetes. As reported previously [11, 12],* D*1 of diabetic patients was significantly smaller than that of control subjects. This has been considered to be due to sympathetic nerve dysfunction [33, 34]. The good correlations observed between* D*1 and CNFD, CNFL, CNBD, CNBL, and the amplitude of the median nerve seem to be compatible with changes in sympathetic nerve function. Hypertension has been reported as a risk factor for diabetic neuropathy [34] and has been associated with small pupil size in type 1 diabetic patients [35]; this is consistent with the present study in that high blood pressure was related to a small* D*1.

Although we employed 470 nm light for elucidating the function of ipRGCs, the ipRGCs have synapses with bipolar and amacrine cells for signaling between the outer and inner retina [36, 37], which are thought to modulate light detection in the PLR. Therefore, the PLR stimulated by 470 nm light should be considered to represent the function of ipRGCs modified by outer retinal signals. However, because we did not perform spectral analysis of PLR kinetics, it was not possible for the present study to exactly determine the relative contributions of cones-rods and ipRGCs to the parameters of the PLR.

*T*1 obtained with blue light of a high intensity and long irradiation period (>5–10 sec) is longer than that occurring with red light [4, 8]. There has been no study comparing* T*1 with blue or red light for 1 or 2 sec or comparing healthy people and diabetic patients.* T*1 of diabetic patients occurring with nonchromatic light irradiation was reported to be significantly longer than that occurring in control subjects and correlated with thermal PTs and glycemic control [38]. In the present study, in control subjects and diabetic patients,* T*1 with 470 nm light was shorter than that with 635 nm light, and in the diabetic patients, the* T*1 with blue light was positively related to age and HbA1c level and inversely associated with HDL-cholesterol level. As the efferent loop of the PLR (sympathetic and parasympathetic nerve) is the same, 470 nm light seemed to evoke a stronger signal than 635 nm light in ipRGCs transmitting to the olivary pretectal nucleus. HDL-cholesterol plays an important role in the functions of RGCs [39], so the HDL-cholesterol levels appeared to favorably influence the PLR kinetics.

Park et al. [31] compared PLRs using blue and red stimuli of different intensities for 1 sec in normal subjects and reported that low-intensity blue stimulation induced larger PC, which was sustained longer after light offset compared with that caused by red stimulus. These results were similar to those of the present study. Feigl et al. [13], using 10 sec chromatic stimulation, reported that the transient kinetics of PC caused by blue stimulation were within the normal range in type 2 diabetic patients. In contrast, the present study revealed that the constriction kinetics caused by blue light in diabetic patients were significantly smaller and slower than those caused in control subjects. This difference may be due to the duration and intensity of light used. Furthermore, Feigl et al. matched the intensity of irradiance between control subjects and diabetic patients [13], while we did not. The present study revealed that the PIPR after blue light offset (mediated solely by ipRGCs at ≥1.7 sec and by rods and ipRGCs at <1.7 sec) in diabetic patients, irrespective of neuropathy severity, was significantly smaller than that of the control subjects. However, there was no difference in PIPR after red light offset at <1.7 sec and ≥1.7 sec between the control subjects and diabetic patients. This might reflect the compromised function of ipRGCs in patients with diabetes. However, baseline pupil size influences the amplitude of pupil constriction and PIPR after light offset [40]. We normalized pupil diameter constriction ([*D*1 −* D*2]/*D*1 [%]) and PIPR after light offset to* D*1. The percentage constriction caused by blue light in control subjects was significantly larger than that in all diabetic patients (*p* = 0.005) and in the stage II subgroup (*p* = 0.016). After normalization to* D*1, the PIPR at ≥1.7 sec in the control group was larger than that in the diabetic patient group (*p* = 0.015) and the stage II subgroup (*p* = 0.029), indicating that* D*1 appears to influence the parameters of PLR kinetics to some extent. Therefore, the irradiation level should be equalized between control subjects and diabetic patients to prevent possible false differences due to small pupil size and accelerated lenticular yellowing in diabetic patients [41].

Although we used 20 cd/m^2^ light at 470 nm and 635 nm to assess PLR, photoreceptors (cones, rods, and ipRGCs) respond to light by sensing photon density. According to the equation by the manufacturer, 20 cd/m^2^ light at 470 nm is equivalent to 4.79 × 14 log photons/cm^2^/sec, and 635 nm is equivalent to 2.14 × 14 log photons/cm^2^/sec, when the pupil diameter is assumed to be 6.0 mm. As blue light contains twice the amount of photons as red light, the more intense and rapid PLR kinetics caused by blue light may be due to the larger numbers of photons of the blue light. We therefore also compared the kinetic parameters of the PLR induced by 20 cd/m^2^ blue and 100 cd/m^2^ red lights in control subjects. The latter red light comprises 1.07 × 15 log photons/cm^2^/sec, more than twice the number of 20 cd/m^2^ blue light. The kinetic parameters resulting from this were as follows (blue versus red, resp.):* T*1, 247–260 versus 257–269 msec, *p* < 0.05; PC, 2.32–2.38 versus 1.95–2.17 mm, *p* < 0.01; CS, 2.01–2.18 versus 1.84–1.99 *μ*/msec, *p* < 0.05; PIPR at ≥1.7 sec, 2778–3450 versus 2241–2762 pixels, *p* < 0.01. The 20 cd/m^2^ blue light had better kinetic parameters than did 100 cd/m^2^ red light. It therefore seemed unlikely that the differences in PLR kinetics were caused by the higher photon density of the blue light.

The present study focused on the dysfunction of the retinal inner and outer photoreceptors as an etiology of impaired PLR caused by diabetes. Of course, the PLR is the results of a neural reflex that is dependent upon pathways and synaptic events beyond the retina. The correlations between the parameters of PLR kinetics and clinical factors, neurophysiological tests, and CNF measures might indicate the disturbance of PLR arc beyond the retinal photoreceptors. Since some CNF measures and the parameters of PLR kinetics were impaired even in diabetic patients without neuropathy, CNF morphology and PLR may be an early clinical predictor of the onset of clinical diabetic neuropathy. Although the size and frequency of beading change in the early stages of hyperglycemia, a fact that is related to impaired peripheral nerve function [20], these factors were not related to the PLR parameters.

We acknowledge that the present study has limitations, which may affect the interpretation of the results. First, although we used 470 nm and 635 nm light to differentially elucidate the functions of ipRGCs and the outer retina, the ipRGCs receive synaptic signals from the outer retina. The relative contributions of cones-rods and ipRGCs to PLR parameters change depending on the magnitude and period of irradiation. We did not perform spectral analysis. Therefore, the present study was not able to assess the relative contribution of ipRGCs to blue light (470 nm) induced PLR kinetic parameters. Second, we did not dilate the pupils, and the pupil diameter of the diabetic patients was smaller than that of the control subjects. We excluded subjects older than 55 years because of potential age-related yellowing of the lens, but we did not perform any specific ophthalmological examinations for diseases of the cornea or lens. The lenses of diabetic patients become yellow at an accelerated rate compared with that of healthy people [41]. It was not possible to exclude the potential influence of these experimental conditions on the attenuation of blue light, resulting in an impaired PLR evoked by blue light in diabetic patients. Third, even though 20 cd/m^2^ blue light induced better PLR kinetics than did 100 cd/m^2^ red light, the photon densities of 470 nm and 635 nm light stimuli would ideally be matched.

We acknowledge that the pupillometry protocol that applied at this work is not a sensitive measure of melanopsin function, and the protocol has limited capacity to detect melanopsin dysfunction in the diabetic patients.

Finally, although some clinical factors were significantly associated with PLR parameters, clarifying the meaning of these relationships was beyond the scope of the present study. Unfortunately, we were not able to find clinical factors that contributed to impaired ipRGC function in the PIPR in diabetic patients.

## 5. Conclusions

In conclusion, this study confirmed in a large population of patients with type 2 diabetes that PIPR is impaired after blue light offset, as previously reported by Feigl et al. [13]. The novel finding from this study is that blue light induced a more intense and rapid PLR in control subjects and diabetic patients than did red light, and the PLR stimulated by blue light in type 2 diabetic patients without DAN was more severely impaired than that caused by red light. It is therefore possible to detect ipRGCs dysfunction before the development of DAN. However, refined methods are required to confirm these results.

## Figures and Tables

**Table 1:** Clinical characteristics in control subjects and subgroups of type 2 diabetic patients stratified by the severity of diabetic neuropathy.

	Control subjects	Type 2 diabetic patients staged by neuropathy severity		
		Stage I	Stage II	Stage III
Number (M/F, M%)	42 (27/15, 64.3 )	31 (20/11, 64.5)	38 (25/13, 65.8)	34 (22/12, 64.7)
Age (years)	41.1–46.2	42.9–47.6	43.5–48.5	43.4–49.1
BMI (kg/m^2^)	21.8–23.6	24.1–29.8^*∗*^	24.8–27.8^†^	26.8–29.8^†^
Systolic blood pressure (mmHg)	120.5–127.3	129.9–139.4^‡^	133.2–142.7^†^	136.6–147.3^†^
Diastolic blood pressure (mmHg)	75.5–79.0	78.2–84.7	78.9–84.5	81.8–89.4^†^
Number treated with angiotensin receptor blocker (%)	2 (4.8)	13 (41.9)^†^	15 (39.5)^†^	8 (23.5)^‡^
HbA1c (%, NGSP)	5.4–5.6	6.4–6.9^†^	6.9–8.3^†^	7.7–9.3^†,§^
HbA1c (mmol/mol)	35.4–37.5	46.7–52.0	52.4–67.5	60.7–78.6
Low density lipoprotein-cholesterol (mmol/L)	2.91–3.42	3.06–3.78	3.24–3.83	3.34–3.94
Number treated with statins (%)	3 (7.1)	2 (6.5)	4 (10.5)	5 (14.7)
High density lipoprotein-cholesterol (mmol/L)	1.59–1.84	1.17–1.48^*∗*^	1.25–1.56^*∗*^	1.23–1.52^*∗*^
Triglycerides (mmol/L)	1.11–1.84	1.43–2.07	1.32–2.84	1.77–2.65^*∗*^
ACR (mg/gCr)	5.1–12.2	0.0–31.5	10.0–24.2	19.4–193.8^*∗*^
eGFR (ml/min)	78.6–88.2	78.9–92.1	80.0–91.9	78.8–90.5
Diabetic retinopathy (no/simple, %/%)		28/3, 90.3/9.7	33/5, 86.8/13.2	27/7, 79.4/20.6
Duration of diabetes (years)		4.1–8.1	5.4–9.6	5.3–10.6

Data are the 95% confidence intervals (CI) in control subjects and the subgroups of the type 2 diabetic patients stratified by the stages of the neuropathy according to the criteria of the Diabetic Neuropathy Study Group in Japan [14]. ^*∗*^*p* < 0.01 compared with control subjects, ^†^*p* < 0.001 compared with control subjects, ^‡^*p* < 0.05 compared with control subjects, and ^§^*p* < 0.01 compared with patients at stage I neuropathy.

ACR: urinary albumin/creatinine ratio; BMI: body mass index; eGFR: estimated glomerular filtration rate.

**Table 2:** Neurophysiological functions in control subjects and subgroups of type 2 diabetic patients stratified by the severity of neuropathy.

	Control subjects	Type 2 diabetic patients staged by neuropathy severity		
		Stage I	Stage II	Stage III
MCV of median nerve (m/sec)	57.3–59.8	55.6–57.6	54.1–56.1^*∗*^	50.4–53.6^†,‡^
Amplitude of median nerve (mV)	6.68–8.94	5.85–8.74	4.24–5.82^*∗*^	2.82–5.15^†,‡^
SCV of ulnar nerve (m/sec)	62.8–65.1	61.0–63.5	60.1–62.3^*∗*^	57.0–59.7^†,‡^
Amplitude of ulnar nerve (*µ*V)	31.5–41.2	22.3–32.4	20.6–28.2^*∗*^	15.5–22.8^†,‡^
SCV of sural nerve (m/sec)	47.0–49.1	46.5–49.9	46.4–49.3	45.2–48.0^§^
Amplitude of sural nerve (*µ*V)	11.5–14.1	9.75–15.1	8.8–11.9	8.5–11.5
Vibration perception threshold (*µ*/120 c/sec)	1.56–2.64	1.76–3.62	1.91–3.15	2.30–4.00
CV_R-R_ (%)	3.74–4.45	3.41–4.56	3.51–4.57	2.85–3.98
Warm perception threshold (W/m^2^)	−602–−496	−582–−465	−619–−517	−616–−512
Cold perception threshold (W/m^2^)	473–588	443–530	509–591	493–586

Data are the 95% confidence intervals in control subjects and the subgroups of the type 2 diabetic patients stratified by the stages of the neuropathy according to the criteria of the Diabetic Neuropathy Study Group in Japan [14]. ^*∗*^*p* < 0.01 compared with control subjects, ^†^*p* < 0.001 compared with control subjects, ^‡^*p* < 0.01 compared with patients at stage I neuropathy, and ^§^*p* < 0.05 compared with control subjects.

CV: coefficient of variation; MCV: motor nerve conduction velocity; SCV: sensory nerve conduction velocity.

**Table 3:** Corneal nerve fiber measures in control subjects and subgroups of type 2 diabetic patients stratified by the severity of neuropathy.

	Control subjects	Type 2 diabetic patients staged by neuropathy severity		
		Stage I	Stage II	Stage III
Corneal nerve fiber density (no/mm^2^)	30.6–34.1	25.6–29.4^*∗*^	24.9–28.1^†^	21.6–25.7^†^
Corneal nerve fiber length (mm/mm^2^)	12.1–13.3	10.6–12.0	10.1–11.5^*∗*^	8.95–10.4^†,‡^
Corneal nerve branch density (no/mm^2^)	12.0–15.6	10.7–13.0	8.7–11.2^*∗*^	8.4–11.0^*∗*^
Corneal nerve branch length (mm/mm^2^)	2.43–3.08	2.31–2.78	2.03–2.64	2.01–2.75
Tortuosity grade	1.86–2.04	2.40–2.64^†^	2.39–2.58^†^	2.47–2.68^†^
Beading frequency (no/0.1 mm)	23.5–24.6	19.3–20.7^†^	19.2–20.4^†^	19.5–20.8^†^
Bead size (*µ*m^2^)	7.89–8.25	9.80–10.2^†^	9.90–10.3^†^	10.0–10.4^†^

Data are the 95% confidence intervals in control subjects and the subgroups of the type 2 diabetic patients stratified by the stages of the neuropathy according to the criteria of the Diabetic Neuropathy Study Group in Japan [14]. ^*∗*^*p* < 0.01 compared with control subjects, ^†^*p* < 0.001 compared with control subjects, and ^‡^*p* < 0.05 compared with patients at stage I neuropathy.

**Table 4:** Comparison of the parameters of pupillary light reflex between control subjects, type 2 diabetic patients, or their subgroups stratified by the severity of neuropathy.

	Baseline pupil size (mm)	Latency period (msec)	Time for minimal pupil size (msec)	Pupil diameterconstriction (mm)	Pupil constrictionvelocity (*µ*/msec)	PIPR (pixels) 0–1.7 sec ≥ 1.7–3.0 sec	
*Control subjects*							
470 nm	5.70–5.92	247–260^*∗*^	1096–1144	2.25–2.47^*∗*^	2.01–2.18^*∗*^	6862–7779^*∗*^	2739–3407^*∗*^
635 nm	5.63–5.90	285–297	848–971	1.43–1.72	1.62–1.85	3942–5009	1337–1920
*Total type 2 diabetic patients*							
470 nm	5.14–5.36^†^	267–276^*∗*,†^	1049–1100	1.83–2.04^*∗*,†^	1.69–1.85^‡,†^	5620–6301^*∗*,†^	2047–2525^*∗*,†^
635 nm	5.17–5.46^†^	301–313^†^	806–889	1.27–1.46^†^	1.53–1.70	3474–4178	1245–1614
*Subgroups of neuropathy severity*							
Stage I							
470 nm	5.29–5.63^§^	263–280^§,*∗*^	1048–1132	1.81–2.22^||,*∗*^	1.66–1.97^||,‡^	5360–6693^||^	1958–2913^§^
635 nm	5.18–5.73^§^	300–324^||^	754–922	1.15–1.51	1.44–1.73	3022–4289	1023–1694
Stage II							
470 nm	5.02–5.37^†^	262–279^§,*∗*^	1028–1108	1.67–2.01^†,*∗*^	1.56–1.83^†,‡^	5375–6290^†^	1699–2452^†^
635 nm	5.04–5.51^§^	295–312	811–930	1.26–1.54	1.49–1.74	3483–4536	1176–1695
Stage III							
470 nm	4.88–5.35^†^	265–279^§,*∗*^	1013–1120	1.77–2.16^||,*∗*^	1.67–1.94^||,¶^	5333–6749^||^	1948–2811^||^
635 nm	4.94–5.52^§^	295–316^||^	748–911	1.17–1.54	1.45–1.82	3058–4508	1096–1880

Data are the 95% confidence intervals (CI) in control subjects, total type 2 diabetic patients, and their subgroups stratified by the stages of the neuropathy according to the criteria of the Diabetic Neuropathy Study Group in Japan [14]. ^*∗*^*p* < 0.001 compared with 635 nm light, ^†^*p* < 0.001 compared with control subjects, ^‡^*p* < 0.01 compared with 635 nm light, ^§^*p* < 0.05 compared with control subjects, ^||^*p* < 0.01 compared with control subjects, and ^¶^*p* < 0.05 compared with 635 nm. PIPR: postillumination pupillary response.

**Table 5:** Relationship between the parameters of pupillary light reflex and clinical factors, neurophysiological tests, or corneal nerve fiber measures in total type 2 diabetic patients.

	Baseline pupil diameter		Latency period				Pupil diameter constriction				Pupil constriction velocity			
			470 nm		635 nm		470 nm		635 nm		470 nm		635 nm	
	St. *β*	*p*	St. *β*	*p*	St. *β*	*p*	St. *β*	*p*	St. *β*	*p*	St. *β*	*p*	St. *β*	*p*
Age	−0.177	0.100	0.245	**0.025**	0.063	0.570	−0.255	**0.020**	−0.236	**0.032**	−0.237	**0.041**	−0.087	0.435
SBP	−0.263	**0.010**	0.034	0.739	0.126	0.232	−0.185	0.072	−0.109	0.287	−0.140	0.178	−0.009	0.933
DBP	−0.324	**0.002**	−0.039	0.707	0.088	0.411	−0.118	0.262	−0.149	0.153	−0.083	0.433	−0.072	0.502
HbA1c	−0.157	0.159	0.238	**0.035**	−0.030	0.797	−0.057	0.614	0.028	0.808	0.003	0.977	0.071	0.545
HDL-C	−0.116	0.282	−0.275	**0.013**	−0.166	0.143	0.060	0.580	0.201	0.070	0.121	0.275	0.200	0.078
Amplitude of MN	0.231	**0.024**	−0.027	0.794	0.012	0.912	0.180	0.084	0.066	0.530	0.145	0.172	0.024	0.822
CNFD	0.267	**0.006**	0.156	0.115	0.105	0.306	0.077	0.438	0.102	0.308	0.079	0.435	0.102	0.323
CNFL	0.242	**0.014**	0.158	0.113	0.084	0.418	0.072	0.472	0.112	0.265	0.076	0.457	0.105	0.310
CNBD	0.310	**0.001**	−0.057	0.578	0.090	0.395	0.118	0.252	0.182	0.077	0.076	0.468	0.071	0.506
CNBL	0.272	**0.005**	−0.012	0.911	0.070	0.512	0.106	0.308	0.163	0.117	0.064	0.544	0.102	0.339

CNBD: corneal nerve branch density; CNBL: corneal nerve branch length; CNFD: corneal nerve fiber density; CNFL: corneal nerve fiber length, CV: coefficient of variation; DBP: diastolic blood pressure; HDL: high density lipoprotein; LDL: low density lipoprotein; MCV: motor nerve conduction velocity; MN: median nerve; SBP: systolic blood pressure; St.: standard.
